# Purification and characterization of bacteriocins-like inhibitory substances from food isolated *Enterococcus faecalis* OS13 with activity against nosocomial enterococci

**DOI:** 10.1038/s41598-021-83357-z

**Published:** 2021-02-15

**Authors:** Ahmed O. El-Gendy, Dag A. Brede, Tamer M. Essam, Magdy A. Amin, Shaban H. Ahmed, Helge Holo, Ingolf F. Nes, Yara I. Shamikh

**Affiliations:** 1 Microbiology and Immunology Department, Faculty of Pharmacy, Beni-Suef University, Salah Salem Street, Beni Suef, 62511 Egypt; 2 Department of Chemistry, Biotechnology and Food Science, Laboratory of Microbial Gene Technology and Food Microbiology, Norwegian University of Life Sciences, Ås, Norway; 3 Microbiology and Immunology Department and Biotechnology Centre, Faculty of Pharmacy, Cairo University, Cairo, Egypt; 4 Microbiology and Immunology Department, Faculty of Medicine, Assiut University, Asyût, Egypt; 5 Department of Microbiology and Immunology, Nahda University, Beni Suef, Egypt; 6 Department of Virology, Egypt Center for Research and Regenerative Medicine, Cairo, Egypt

**Keywords:** Microbiology, Applied microbiology

## Abstract

Nosocomial infections caused by enterococci are an ongoing global threat. Thus, finding therapeutic agents for the treatment of such infections are crucial. Some *Enterococcus faecalis* strains are able to produce antimicrobial peptides called bacteriocins. We analyzed 65 *E. faecalis* isolates from 43 food samples and 22 clinical samples in Egypt for 17 common bacteriocin-encoding genes of *Enterococcus* spp. These genes were absent in 11 isolates that showed antimicrobial activity putatively due to bacteriocins (three from food, including isolate OS13, and eight from clinical isolates). The food-isolated *E. faecalis* OS13 produced bacteriocin-like inhibitory substances (BLIS) named enterocin OS13, which comprised two peptides (enterocin OS13α OS13β) that inhibited the growth of antibiotic-resistant nosocomial *E. faecalis* and *E. faecium* isolates*.* The molecular weights of enterocin OS13α and OS13β were determined as 8079 Da and 7859 Da, respectively, and both were heat-labile. Enterocin OS13α was sensitive to proteinase K, while enterocin OS13β was resistant. Characterization of *E. faecalis* OS13 isolate revealed that it belonged to sequence type 116. It was non-hemolytic, bile salt hydrolase-negative, gelatinase-positive, and sensitive to ampicillin, penicillin, vancomycin, erythromycin, kanamycin, and gentamicin. In conclusion, BLIS as enterocin OS13α and OS13β represent antimicrobial agents with activities against antibiotic-resistant enterococcal isolates.

## Introduction

Lactic acid bacteria (LAB) are defined as aerotolerant Gram-positive cocci or bacilli that ferment sugars to produce lactic acid^1,2^. Enterococci are LAB, and they are found in the gastrointestinal tract of humans and animals as well as in feed, food, and healthcare units^3^. Most strains of *Enterococcus faecalis* are harmless commensals that may be beneficial to health (probiotics). In contrast, other strains are opportunistic pathogens, especially vancomycin-resistant enterococci (VRE) and multidrug-resistant enterococci (MDRE), causing nosocomial infections and acquiring antibiotic resistance determinants that become intrinsically resistant to commonly used antibiotics, leading to an increasingly serious problem in public health worldwide^4^. *E. faecalis* and *E. faecium* are recognized as the most common causes of nosocomial infections in their genus, causing urinary tract infection, postoperative wound infection, endocarditis, bacteremia, pneumonia, and meningitis^5^. The search for novel effective antimicrobial agents has become a global concern due to the increasing prevalence of MDRE and VRE. Over the past three decades, the rate of antibiotic discovery has slowed, and few antibiotics have been developed against VRE^6^. Furthermore, some enterococci have evolved resistance to the most recently-developed antimicrobial agents, such as quinupristin-dalfopristin, linezolid, daptomycin, and tigecycline^7^.

Antimicrobial peptides (AMPs) have drawn attention as alternatives to conventional antibiotics. Enterococci produce a variety of ribosomally-synthesized AMPs or bacteriocins^8–10^. There are three classes of bacteriocins. Briefly, class I consists of small, heat-stable, post-translationally modified peptides. Lantibiotics, due to the presence of the modified residues lanthionine and methyl-lanthionine, are a well-known example of that class. Some other bacteriocins are also considered members of class I, namely, those belonging to ribosomally-synthesized and post-translationally modified peptides that function as bacteriocins, such as circular bacteriocins, sactibiotics, and glycocins^11^. Class II consists of small, heat-stable peptides without modified residues. Class III consists of abundant, heat-labile proteins^12,13^.

Bacteriocins have been used in the food industry for over 50 years and are Generally Recognized As Safe (GRAS)^14–16^. The use of bacteriocins as a replacement for currently used antibiotics is promising^15^. Furthermore, resistance toward bacteriocins has rarely been observed^17^. Because enterococci are part of the healthy microbiota in humans, they are considered relatively safe but are not endowed with GRAS status^18^. However, some of them are used as probiotics^19^, and bacteriocin production adds an essential feature in their protection against pathogenic bacteria^20^.

Bacteriocins differ from antibiotics in that they are ribosomally synthesized and target bacteria closely related to the producing strain by different mechanisms of action^12^. These killing mechanisms include membranous pore formation, disruption of the cell wall or protein synthesis, and degradation of nucleic acids^21^. Narrow-spectrum antibacterials, such as bacteriocins, are an attractive approach in the battle against multidrug-resistant bacterial pathogens due to their limited resistance acquisition and trigger no significant disruption to the host microbiota. It worth mentioning that the use of traditional broad-spectrum antibiotics can disturb the host microbiota, affecting the crucial role that it plays in various functions, including nutrient and certain amino acids supply, vitamin production, and protection against pathogens^22^. Exposure to broad-spectrum antibiotics during infancy and early childhood is mostly unfavorable, as the early microbiota lacks diversity and stability, making it distinctively sensitive to disruption, besides the importance of the gut microbiota in the early development and education of the immune system^23^. Thus, if the infection's causative pathogen is well-known, the use of narrow-spectrum antimicrobials, such as bacteriocins, could potentially avoid these issues of hurting gut microbiota.

In these perspectives, the main objective of this study was to screen different *E. faecalis* isolates, obtained from food and clinical samples, for their ability to produce bacteriocins. Attempts were performed to purify and characterize BLIS with activity against nosocomial enterococci. One putative bacteriocinogenic isolate was studied in detail, and two BLIS were characterized from this isolate.

## Materials and methods

### Bacterial strains and culture conditions

The bacterial strains used in this study and their culture conditions are presented in Table 1. All strains were maintained in glycerol stock 40% (v/v) and stored at − 80 ºC.

**Table 1:** Inhibition spectrum of BLIS from *E. faecalis* isolates using a deferred inhibition test method.

Indicator strain^a^	Description	Growth medium^b^	Growth temp (°C)	Bacteriocin producers; inhibition^c^										
				OS7	OS11	OS13	OS16	OS28	OS29a	OS29b	OS62a	OS62b	OS64a	OS65
*Asperigellus niger* ATCC 16888	Tested indicator strain	SDA	30	**–**	**–**	**–**	**–**	**–**	**–**	**–**	**–**	**–**	**–**	**–**
*Bacillus subtilis* subsp. *spizizenii* ATCC 6633	Tested indicator strain	BHI	37	**–**	**–**	**–**	**–**	**–**	**–**	**–**	**–**	**–**	**–**	**–**
*Candida albicans* ATCC 60193	Tested indicator strain	SDA	30	**–**	**–**	**–**	**–**	**–**	**–**	**–**	**–**	**–**	**–**	**–**
*Enterococcus faecalis* LMG 2708	Pediocin sensitive strain	GM17	30	**–**	**–**	**–**	**–**	**–**	**–**	**–**	**–**	**–**	**–**	**–**
*Enterococcus faecalis* LMG 2708 *R	Pediocin resistant mutant	GM17	30	**–**	**–**	**–**	**–**	**–**	**–**	**–**	**–**	**–**	**–**	**–**
*Enterococcus faecalis* LMG 2333	Source of enterolysin A	GM17	30	**–**	**–**	** + **	**–**	**–**	**–**	**–**	**–**	**–**	**–**	**–**
*Enterococcus faecalis* LMG 2814	Source of cytolysin	GM17	30	**–**	**–**	** + **	**–**	**–**	**–**	**–**	**–**	**–**	**–**	**–**
*Enterococcus faecalis OS 63*	Nosocomial multi-resistant isolate	GM17	30	**–**	**–**	** + **	**–**	**–**	**–**	**–**	**–**	**–**	**–**	**–**
*Enterococcus faecium* LMG 2771	Source of enterocin A and enterocin B	GM17	30	**–**	**–**	** + **	**–**	**–**	**–**	**–**	**–**	**–**	**–**	**–**
*Enterococcus faecium* LMG 2772	Source of enterocin P	GM17	30	**–**	**–**	** + **	**–**	** + **	** + **	** + **	** + **	** + **	** + **	** + **
*Enterococcus faecium* LMG 2763	Source of enterocin Q, enterocin L50A and L50B	GM17	30	**–**	**–**	** + **	**–**	**–**	**–**	**–**	**–**	**–**	**–**	**–**
*Enterococcus faecium* AT 122	Nosocomial multi-resistant isolate	GM17	30	**–**	**–**	** + **	**–**	**–**	**–**	**–**	**–**	**–**	**–**	**–**
*Enterococcus faecalis* V583	Nosocomial multi-resistant isolate	GM17	30	**–**	**–**	** + **	**–**	**–**	**–**	**–**	**–**	**–**	**–**	**–**
*Escherichia coli* ATCC 5087	Tested indicator strain	BHI	37	**–**	**–**	**–**	**–**	**–**	**–**	**–**	**–**	**–**	**–**	**–**
*Lactobacillus plantarum* LMG 2003	Tested indicator strain	MRS	37	** + **	** + **	** + **	** + **	** + **	** + **	** + **	** + **	** + **	** + **	** + **
*Lactobacillus sakei* LMG 2313	Tested indicator strain	MRS	30	** + **	** + **	** + **	** + **	** + **	** + **	** + **	** + **	** + **	** + **	** + **
*Listeria innocua* LMG 2710	Pediocin sensitive strain	GM17	37	**–**	**–**	**–**	**–**	**–**	**–**	**–**	**–**	**–**	**–**	**–**
*Pseudomonas aeruginosa* ATCC 9027	Tested indicator strain	BHI	37	**–**	**–**	**–**	**–**	**–**	**–**	**–**	**–**	**–**	**–**	**–**
*Salmonella enterica* ATCC 35664	Tested indicator strain	BHI	37	**–**	**–**	**–**	**–**	**–**	**–**	**–**	**–**	**–**	**–**	**–**
*Staphylococcus aureus* subsp. *aureus* LMG 3240	Non-pathogenic strain	BHI	37	**–**	**–**	**–**	**–**	**–**	**–**	**–**	**–**	**–**	**–**	**–**
*Staphylococcus aureus* subsp. *aureus* LMG 3242	Pathogenic strain	BHI	37	**–**	**–**	**–**	**–**	**–**	**–**	**–**	**–**	**–**	**–**	**–**
*Staphylococcus aureus* subsp. *aureus* ATCC 6538	Tested indicator strain	BHI	37	**–**	**–**	**–**	**–**	**–**	**–**	**–**	**–**	**–**	**–**	**–**

^a^ATCC, “American Type Culture Collection”.

^b^SDA, “Sabouraud Dextrose Agar”; BHI, “Brain Heart Infusion”; MRS, “deMan, Rogosa and Sharpe”; GM17, “Glucose-M17”.

^c^ + , clear inhibition zones of 12 mm or more were recorded as a positive antibacterial activity.

– , no inhibition.

### Isolation and identification of *E. faecalis*

Different food samples (milk products; mozzarella cheese and buttermilk—pastries; commercial cakes and bread) and clinical samples (urine and stool) were collected in sterile plastic cups from Beni-Suef City, Egypt, and refrigerated for 24 h prior to processing. Urine sample precipitates (collected after centrifugation at 5000×*g* for 10 min) and 1 g of each of the food and stool samples were inoculated into tubes containing 5 mL of GM17 broth (Oxoid, Hampshire, UK) and incubated aerobically at 37 °C for 24 h.

Enterococci were isolated by selective culture on bile esculin agar (Oxoid), where they grow in the presence of 4% (w/v) bile and hydrolyze esculin to esculetin that reacts with Fe^3+^ to form a dark brown to black precipitate on the agar plate. The resultant black colonies were presumptively identified as *Enterococcus* sp. using the l-pyrrolidonyl-β-naphthylamide (PYR) test and then identified at species level using the API 50 CHL system (bioMérieux, Marcy l’Etoile, France) according to the manufacturer’s instructions.

### Genomic DNA isolation

It was performed with some modifications of a previously described protocol^24^. Briefly, 1.5 ml of an overnight culture was centrifuged for 10 min at 3000×*g*, and the supernatant was discarded. The pellet was resuspended in 200 μL of spheroplast buffer (10% [w/v] sucrose, 25 mM Tris [pH 8.4], 25 mM EDTA [pH 8.0], 2 mg/mL lysozyme, 1 mg/mL proteinase K, and 0.4 mg/mL RNase A), vortexed, and incubated at 37 ºC for 10 min until cell lysis occurred. Then, 50 μL of each of 5% (w/v) sodium dodecyl sulfate (SDS) (lysis buffer 1) and 5 M NaCl (lysis buffer 2) were added, mixed, and incubated at 65 ºC for 5 min. Next, 100 μL of neutralizing buffer (60 mL 5 M potassium acetate, 11.5 mL glacial acetic acid, and 28.5 mL dH_2_O) was added. The sample was vortexed, placed on ice for 5 min, and centrifuged at 18,000×*g* at 4 ºC for 15 min. The supernatant (approximately 400 μL) was transferred to a new tube, mixed with an equal volume of isopropanol, left to stand for 5 min at room temperature, and centrifuged at 18,000×*g* at room temperature for 15 min to precipitate the DNA. The resultant pellet was washed with 70% (v/v) ethanol by centrifugation at 18,000×*g* at room temperature for 5 min and finally air-dried, resuspended in 50 μL 1× TE buffer [pH 8], and stored in a refrigerator at 4 ºC. The DNA purity and concentration were determined by optical density measurement using a NanoDrop ND1000 spectrophotometer (Thermo Scientific, Wilmington, DE, USA).

### Polymerase chain reaction (PCR) amplification and sequencing of *16S rDNA* gene

These were accomplished using primers 11F and 4R (Table S1). The PCR amplicon of interest was detected by agarose gel electrophoreses^25^ using 1% (w/v) agarose gels with reference to 1 kbp DNA ladder (Fermentas, Vantaa, Finland). Any impurities were removed from the PCR product using the QIAquick PCR purification kit (Qiagen, Hilden, Germany). Quantification of the PCR products was determined by optical density measurement using a NanoDrop ND1000 spectrophotometer (Thermo Scientific, Wilmington, DE, USA). The PCR product was sequenced and analyzed using an ABI Prism BigDye Terminator v3.1 Ready Reaction Cycle Sequencing Kit on an ABI Prism 3100 Genetic Analyzer, according to the manufacturer’s instructions. The sequences were assembled by BioEdit v7.0.9.0 software, and a homology search of the National Center for Biotechnology Information (NCBI) database was performed using BLAST. Multiple sequence alignment was performed, and molecular phylogenies were evaluated using MEGA7 software using the neighbor-joining method, and the evolutionary distances were computed using the Kimura two-parameter method^26^.

### PCR screening for bacteriocin structural genes

The presence of genes encoding 17 known bacteriocins in *Enterococcus* spp. were determined by PCR amplification using specific primers purchased from Invitrogen (Carlsbad, CA, USA) (Table S1). DNA samples from different standard strains belonging to Laboratory of Microbiology, Department of Biochemistry and Microbiology, Faculty of Sciences of Ghent University, Ghent, Belgium, (LMG) culture collections with known bacteriocin genes were used as positive controls for the PCR reactions (Table 1). The PCR product of interest was detected by agarose gel electrophoreses^25^ using 1.5% (w/v) agarose gels with reference to a 100-bp DNA ladder (Fermentas).

### Screening for antimicrobial activity production

The antimicrobial activity was detected using a deferred inhibition test^27^. Briefly, the colonies were overlaid with 5 mL of soft agar containing 70 µl of the indicator strains which were grown to late exponential phase (Table 1). A confirmatory test was established by adding aliquots of the pH-adjusted supernatant (50 µl) into 0.5-cm wells, made in the inoculated agar plates with indicator strain. Then, the plates were incubated overnight and checked for the presence of growth inhibition zones.

Quantitative estimation of BLIS in the cell-free culture supernatant was performed using a microtiter plate assay^28^, where 100× dilutions of the sensitive indicator strains were exposed to twofold serial dilutions of bacteriocin in growth media. The plates were incubated overnight, and the growth was measured spectrophotometrically at 620 nm using a microtiter plate reader (iEMS Reader; Labsystems, Helsinki, Finland). One arbitrary activity unit (AU) was defined as the reciprocal of the highest dilution of bacteriocin, causing 50% growth inhibition of the treated indicator strain by measuring growth turbidity inhibition at 620 nm in the microtiter plate reader compared to the untreated control^29^. The bacteriocin activity was calculated according to the following equation; Bacteriocin activity (AU/mL) = (d × 1/m), where “d”; is the used dilution factor and “m” is the highest dilution causing 50% growth inhibition.

### Determination of virulence traits (hemolytic activity, proteolytic gelatinase activity, and bile salt hydrolase activity)

Hemolytic activity, being alpha, beta, or gamma, was determined by streaking putative bacteriocinogenic *E. faecalis* isolates onto a fresh plate of Columbia blood agar (Oxoid) containing 5% (v/v) defibrinated horse blood and incubating at 37 °C for 24 h. The formation of a clear zone around the colonies indicated beta complete hemolytic activity. In contrast, the formation of greenish discoloration surrounding the colonies indicated alpha partial hemolytic activity, and the absence of any hemolysis was referred to as gamma or no hemolysis. Proteolytic gelatinase activity was detected by inoculating *E. faecalis* isolates into tubes containing gelatin medium composed of 0.5% (w/v) peptone, 2% (w/v) gelatin, 0.3% (w/v) beef extract, 0.5% (w/v) NaCl and final pH was adjusted to 7.2. All tubes, including the control (uninoculated), were incubated at 37 °C for 24 h. Gelatin liquefaction observed after keeping the tubes at 4 °C for 3 h, indicated positive gelatinase activity. Putative bacteriocinogenic *E. faecalis* isolates were cultured on both control MRS agar and MRS agar containing 0.5% (w/v) thioglycolate and 0.5% (w/v) sodium glychodeoxycholate (bile salt) to investigate bile salt hydrolase activity. The formation of white precipitate around the colonies indicated bile salt hydrolase activity.

### Antibiotic susceptibility testing and minimum inhibitory concentration (MIC) determination

In order to perform antibiotic susceptibility typing of the 11 putative bacteriocinogenic *E. faecalis* isolates to check whether they are relevant to each other or from different clones, and to overview their biosafety for further applications, the Clinical and Laboratory Standards Institute (CLSI) broth microdilution method in microtiter plates^30^ was used to determine the antibiotic resistance phenotype and MIC of these isolates against 13 antibiotics with different concentration ranges (Table S2), using Mueller–Hinton broth (Oxoid). The microtiter plates were incubated at 37 °C for 20 h before visually determining the MIC, which is the lowest drug concentration that causes complete inhibition of microorganism growth.

### Multilocus sequence typing (MLST)

An MLST scheme based on seven housekeeping genes was performed to investigate the epidemiology and population structure of the 11 putative bacteriocinogenic *E. faecalis* isolates^31^. The PCR primers are listed in Table S1. The allelic profile or sequence type (ST) was determined, according to the *E. faecalis* MLST database (http://efaecalis.mlst.net/)^31^. The ST distribution among all putative bacteriocinogenic *E. faecalis* isolates, included in the MLST database, was evaluated using eBurst v3^32^.

### Purification of BLIS

The BLIS was purified as previously described^33^. Briefly, 2 L of *E. faecalis* OS13 cultivated in MRS broth at 30 °C for 18 h was used for further purification steps*.* After centrifugation at 14,500×*g* for 10 min at 4 °C (Sorvall RC-6; Kendro Laboratory Products, Asheville, NC, USA) and filtration (500 mL Steritop 0.22 µm filter system; Millipore, Dublin, Ireland), the supernatant was subjected to ammonium sulfate precipitation 50% (w/v) and shaken vigorously at 4 °C for 30 min. The protein pellet was harvested by centrifugation at 14,500×*g* for 10 min at 4 °C and then redissolved in 40 mL of distilled water to obtain a 50× concentrated crude BLIS solution. Whenever necessary, the pH was adjusted to 3.5 using 0.1 M HCl.

The crude BLIS solution underwent cation exchange chromatography using a 10-mL SP Sepharose Fast Flow column (GE Healthcare Bioscience, Uppsala, Sweden) pre-washed with 200 mL distilled water and equilibrated with 10 mL sodium acetate (10 mM, pH 5.0). After applying the crude BLIS, the column was washed with 50 mL sodium phosphate buffer (20 mM, pH 6.8) and finally eluted using 20 mL of 1 M NaCl. The BLIS was further purified by reverse-phase chromatography on an ÄKTA FPLC purifier system (Amersham Pharmacia Biotech, Amersham, UK). First, the sample was applied to a Resource RPC 1 mL column (Amersham Pharmacia Biotech) pre-equilibrated with 0.1% (v/v) trifluoroacetic acid (TFA) in water (Buffer A) and eluted using a 30 column volume (CV) linear gradient from 0 to 100% (v/v) isopropanol containing 0.1% (v/v) TFA (Buffer B). Fractions of 2 mL each were collected. The most active fraction (determined by microtiter plate assay) was further diluted in 20 mL 0.1% (v/v) TFA in water and applied to a Sephasil Peptide C8 5-µm ST 4.6/250 column (Amersham Pharmacia Biotech). Elution was performed using 0.5 CV (10% (v/v) Buffer B), 1 CV (10–30% (v/v) Buffer B), 5 CV (30–45% (v/v) Buffer B), and finally 2 CV (45–100% (v/v) Buffer B) at a flow rate of 0.4 mL/min. The peaks were monitored at 214, 254, and 280 nm, and fractions (1 mL each) were collected.

### Sodium dodecyl sulfate–polyacrylamide gel electrophoresis (SDS-PAGE) and mass spectrometry

After cation exchange chromatography, the partially purified BLIS was analyzed by one-dimensional SDS-PAGE (Mini-PROTEAN electrophoresis apparatus; Bio-Rad, Hercules, CA, USA) using a 4% (v/v) stacking gel and 15% (v/v) separating gel. A pre-stained molecular mass protein marker with a range of 7000–70,000 Da was used. The BLIS was separated at 180 V for 45 min, and the gel was divided into two identical vertical parts, one of which was stained and visualized using silver nitrate. The other part was used to identify antibacterial activity by first washing the gel three times with distilled water (40 min/wash) to remove excess SDS. Then, the gel was transferred to an MRS agar plate and overlaid with 10 mL of soft MRS agar containing the indicator microorganism (*L. sakei* LMG 2313) and incubated overnight at 30 °C. The molecular weight of the purified peptide after reverse-phase chromatography was further confirmed with matrix-assisted laser desorption ionization (MALDI)-TOF/MS (Voyager-DE RP; Applied Biosystems, Foster City, CA, USA), as described^34,35^. Briefly, 0.5 μL of the sample was mixed with 0.5 μL of the matrix mixed with 15 mg/mL alpha-cyano-4-hydroxycinnamic acid and deposited on a ground steel MALDI plate (Bruker Daltonics, Billerica, MA, USA). The mass window was adjusted between 4000 and 10,000 Da in the linear positive ion mode, with an acceleration voltage of 25 kV. A peptide mass similarity search was performed using ExPASy with the TagIdent tool (http://www.pdg.cnb.uam.es/cursos/Leon_2003/pages/visualizacion/programas_manuales/spdbv_userguide/us.expasy.org/tools/tagident.html).

### Effect of heat and proteinase K enzyme

The thermal stability of both crude and purified BLIS was determined by heating the samples at 100 ºC for 30 min. Sensitivity toward proteinase K (Sigma-Aldrich, St. Louis, MO, USA) at a final concentration of 1 mg/mL was determined for both the crude and purified BLIS by incubating samples (pH 7.5) with or without enzyme at 37 °C for 60 min. Then, the activity of each sample was compared with the untreated sample using a microtiter plate assay with the indicator strain *L. sakei* LMG 2313.

## Results

### Isolation and identification of *E. faecalis*

**Figure 1:**
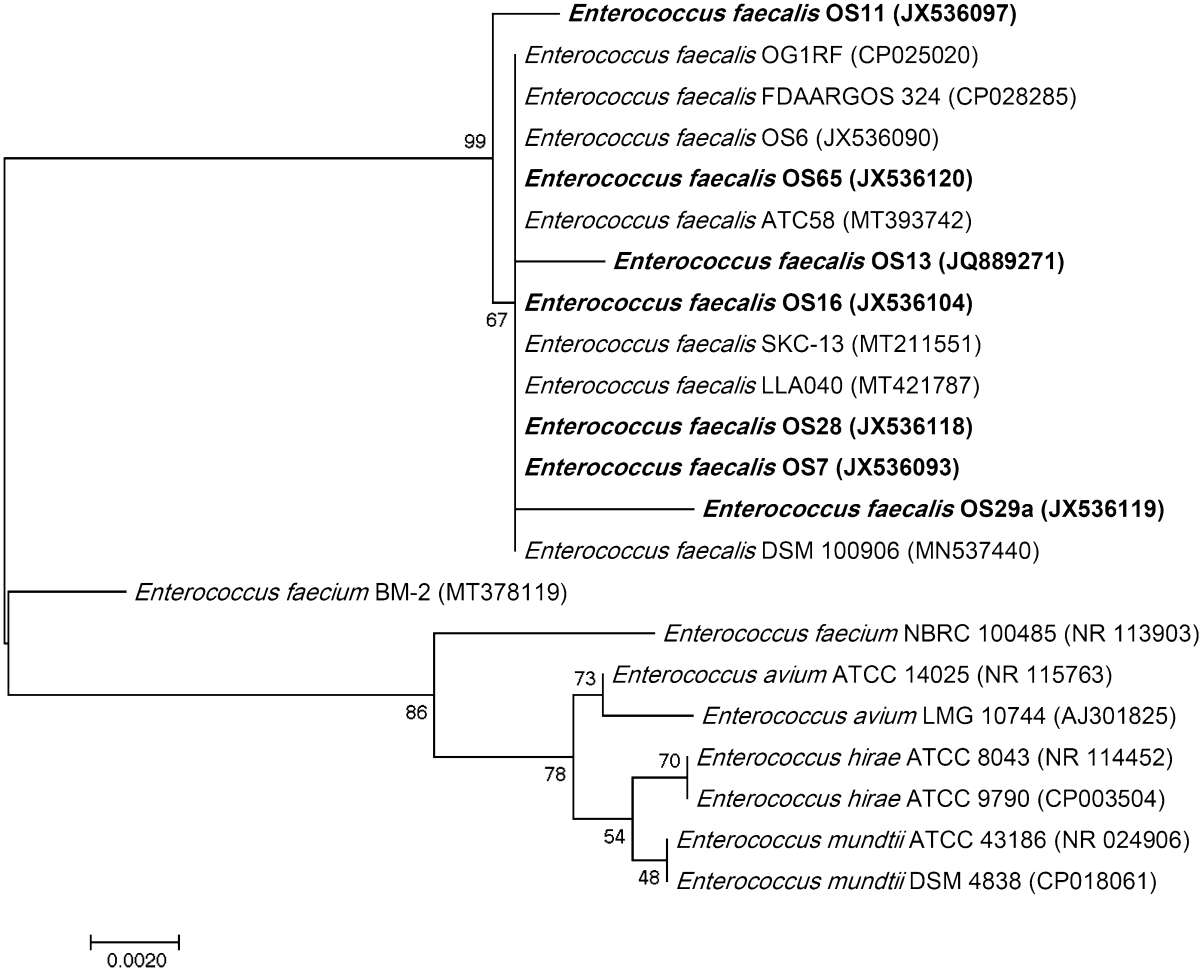
Phylogenetic tree of bacteriocinogenic isolates based on partial 16S rDNA gene sequences. The percentage of replicate trees in which the associated taxa clustered together in the bootstrap test (1000 replicates) are shown next to the branches.

The 65 isolates (43 from food and 22 from clinical samples) that were collected from various food and healthcare units were positive for esculin hydrolysis on bile esculin agar and for l-pyroglutamic acid beta-naphthylamide hydrolysis on PYR discs (results not shown). They were identified at the species level as *E. faecalis* using an API 50 CHL carbohydrate fermentation kit and were positive for glycerol, ribose, galactose, glucose, fructose, mannose, mannitol, sorbitol, N-acetyl glucosamine, amygdalin, arbutin, esculin, salicin, cellobiose, maltose, saccharose, trehalose, melezitose, and tagatose. Furthermore, 16S rDNA sequence analysis confirmed their identity as *E. faecalis*. The obtained sequences of the bacteriocinogenic isolates were submitted to the NCBI GenBank database under specific accession numbers (Table S3). The partial 16S rRNA gene sequences were aligned to closely related species retrieved from the NCBI GenBank database and assembled in MEGA7 software to construct a phylogenetic tree (Fig. 1).

### Screening for bacteriocin genes and spectrum of antimicrobial activity

Interestingly, when the putative bacteriocinogenic *E. faecalis* isolates were screened by PCR for genes corresponding to any of 17 known bacteriocin genes, none of these genes were found in 11 of the isolates (three from food samples and eight from clinical samples). Of them, the food-isolated *E. faecalis* OS13 produced the most potent antimicrobial activity and was chosen for further study and purification of its biologically active antimicrobial substance. Some Gram-positive bacteria, such as *L. sakei*, *L. plantarum*, *E. faecalis*, and *E. faecium* were sensitive to the antimicrobial activity of the OS13 isolate. Still, other Gram-positive bacteria, such as *Listeria*, *Bacillus*, and *Staphylococcus*, and all tested Gram-negative bacteria were resistant. The most potent antimicrobial activity was shown against *L. sakei* LMG 2313, which was then used as the primary indicator microorganism during purification.

### Characterization of the isolates producing antimicrobial activities at biosafety and epidemiology levels

All selected 11 isolates with antimicrobial activities demonstrated bile salt hydrolase activity except strains OS11, 13, 16, and 64. Furthermore, all strains were non-hemolytic and gelatinase-positive. They were sensitive to ampicillin and vancomycin (Table S2) but resistant to clindamycin, fusidic acid, apramycin, streptomycin, tetracycline, and polymyxin B (Table S2, Fig. S1). Other antibiotics sensitivities and resistance patterns are listed (Table S2). Clustering based on the resistance pattern of these isolates is presented in Fig. S1. Finally, the multilocus sequence typing (MLST) of seven housekeeping genes^31^ revealed that these isolates belonged to ST 6 and ST116 (Table S3). ST116, representing *E. faecalis* OS13, did not belong to clonal complex (CC) 6 or CC9, which are commonly spread in the hospital environment (Fig. S2).

### Purification of BLIS from *E. faecalis* OS13

**Figure 2:**
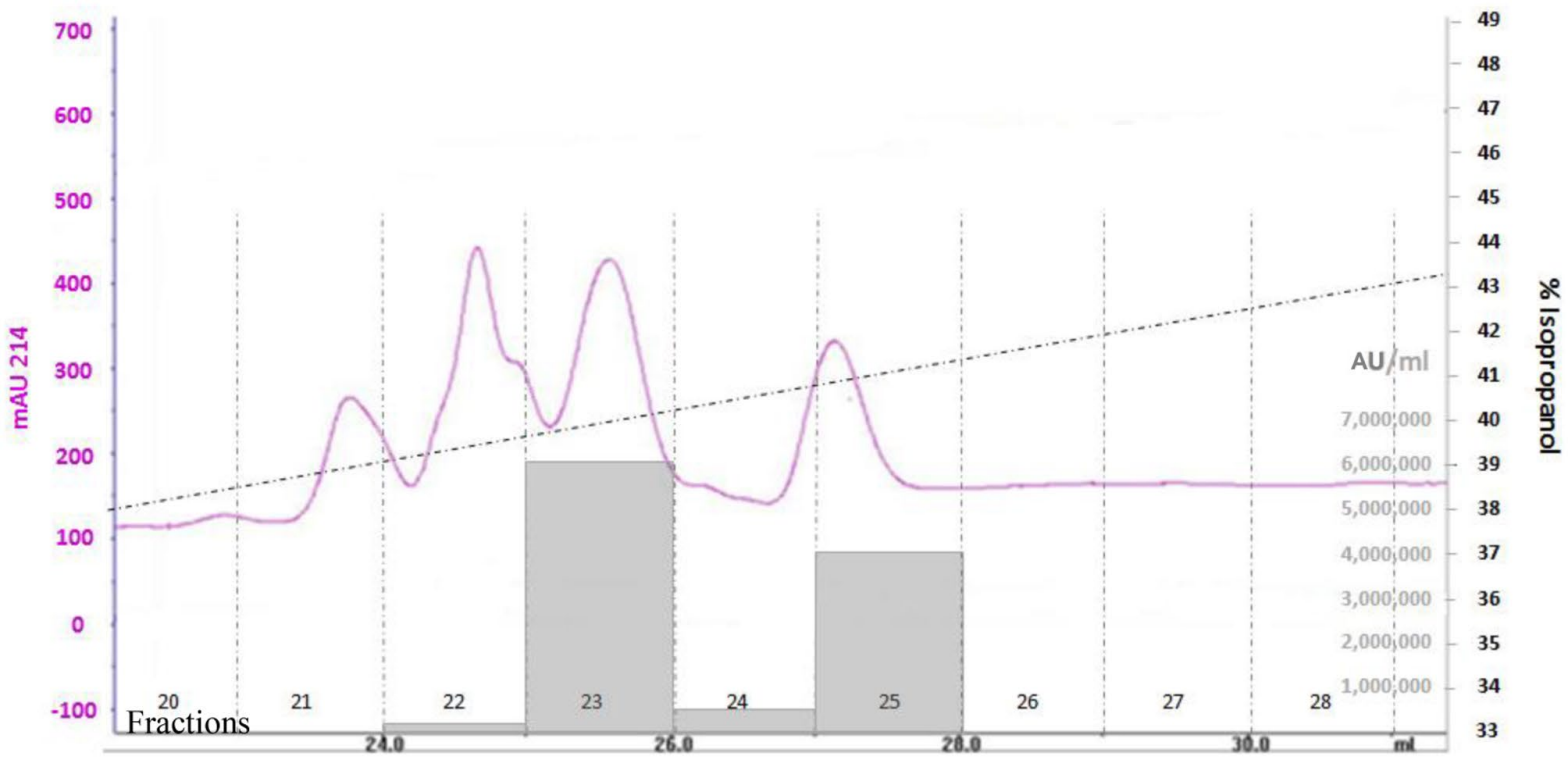
Second reversed-phase chromatography using the Sephasil peptide C_8_ column. This shows two peaks with antimicrobial activity eluted at approximately 39.5% and 40.5% (v/v) isopropanol. The solid line shows the absorbance at 214 nm, while the broken line shows the elution gradient of isopropanol. The antimicrobial activity (AU/mL) is shown as gray columns.

The BLIS produced from isolate OS13 was purified using successive purification steps (Table 2). The activity (AU/mL) was determined by microtiter plate assay using *L. sakei* LMG 2313 as the indicator strain. The final step of reverse-phase chromatography using a Sephasil Peptide C8 column allowed the separation of two peaks with antimicrobial activity, eluted at approximately 39.5% (v/v) and 40.5% (v/v) isopropanol, respectively (Fig. 2).

**Table 2:** Purification of enterocin OS13.

Purification step	Vol (mL)	Activity per mL (AU/mL)^a^	Total activity (AU)	Recovery (%)
Culture supernatant	2000	64,000	128,000,000	100
Ammonium sulfate precipitate	40	4,096,000	163,840,000	128
Ion exchange chromatography	20	2,560,000	51,200,000	40
First reversed-phase chromatography (RPC1 column)	2	12,288,000	24,576,000	19.2
**Second reversed-phase chromatography (sephasil peptide C**_**8**_** column)**				
Enterocin OS13α	1	6,144,000	6,144,000	4.8
Enterocin OS13β	1	4,096,000	4,096,000	3.2

^a^(AU/mL), Arbitrary unit per mL.

### Sodium dodecyl sulfate (SDS)-polyacrylamide gel electrophoresis (PAGE) and mass spectrometry

**Figure 3:**
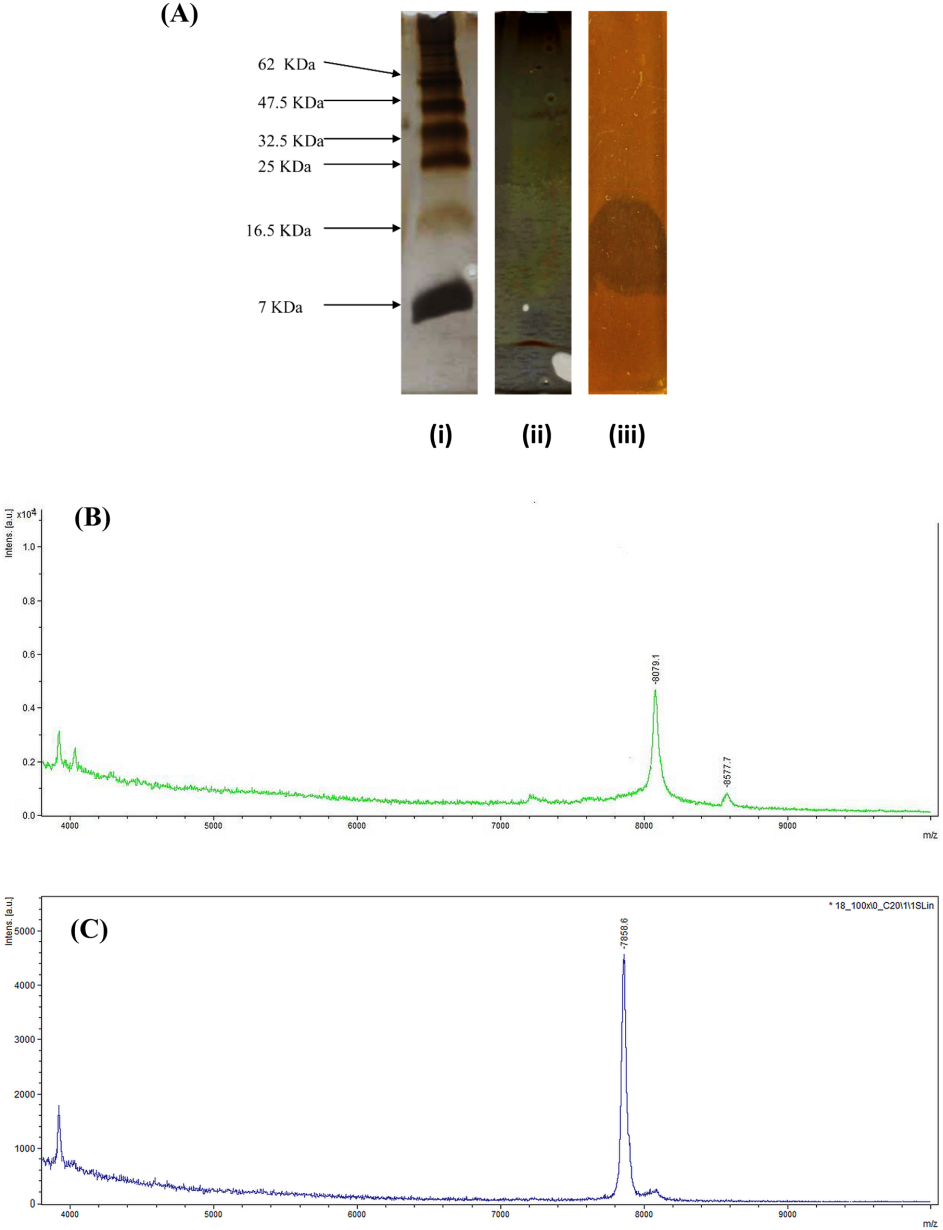
SDS-PAGE (**A**) of protein marker (i) compared to partially purified BLIS after cation exchange chromatography (ii) and showed a single diffused band in the bioassay experiment (iii) with a molecular weight between 7 and 16.5 kDa. The full-length gels are presented in Supplementary Figure S3; (**B**) MALDI-TOV mass spectrometry analysis of enterocin OS13α obtained from second reversed-phase chromatography and (**C**) MALDI-TOV mass spectrometry analysis of enterocin OS13β obtained from second reversed-phase chromatography.

The partially purified BLIS obtained by cation exchange chromatography were analyzed by one-dimensional SDS-PAGE. A single diffuse band with a molecular weight of 7–16.5 kDa was observed that produced a clear zone of inhibition against *L. sakei* LMG 2313 (Fig. 3A). The molecular weights of the purified agents were further determined by MALDI-time of flight mass spectrometry (TOF/MS) analysis. The two BLIS were designated enterocin OS13α and OS13β and showed a molecular weight of 8079.1 Da (Fig. 3B) and 7858.6 Da (Fig. 3C), respectively. A peptide mass similarity search using ExPASy revealed that there were no molecular weight similarities with any other AMPs except holotricin-2 (7858 Da)^36^.

### Effect of heat and proteinase K enzyme

When the crude BLIS with an initial activity of 10,240 AU/mL was heated to 100 °C, the activity was reduced to 640 AU/mL (6.25%). Also, the antimicrobial activity was decreased to 1280 AU/mL (12.5%) after proteinase K treatment. Repeating the same treatment with the concentrated purified enterocin OS13α with initial activity (1,024,000 AU/mL) and enterocin OS13β with initial activity (128,000 AU/mL) confirmed the sensitivity of both peptides to heat treatment, and their activities were entirely abolished. Proteinase K treatment reduced the activity of enterocin OS13α to 512,000 AU/mL (50%), but enterocin OS13β remained unaffected with an activity of 128,000 AU/mL.

## Discussion

*Enterococcus faecalis* are widely spread in the environment and are commonly isolated from foodstuffs and clinical samples^37^. *E. faecalis* and other LAB have attracted more attention over the last few years due to the production of many useful AMPs or BLIS, with several safe applications in the food industry and medical treatments^38,39^. The identification of antimicrobial agents to treat antibiotic-resistant nosocomial enterococci is crucial because the treatment of such infections is becoming increasingly problematic. In addition, enterococci gained greater medical and public attention since the mid-1980s when vancomycin resistance was first present in clinical isolates (VRE)^40^. BLIS with the potential to inhibit the growth of such bacteria could remedy infections without affecting endogenous beneficial microbiota or risking dissemination of R-factors to non-enterococcal pathogens (e.g., vancomycin-intermediate *Staphylococcus aureus*).

Our study searched for suitable candidate BLIS in 65 *E. faecalis* isolates from Egypt of food and clinical origin. The 11 unknown putative bacteriocinogenic *E. faecalis* isolates were characterized epidemiologically by MLST and tested for antibiotic resistance, virulence traits, and antimicrobial activities. Our study showed that, *E. faecalis* OS13 produced highly active (> 10^6^ AU/mL) BLIS designated enterocin OS13α and OS13β that had antimicrobial activity against some Gram-positive bacteria, such as *L. sakei*, *L. plantarum*, *E. faecalis*, and *E. faecium*, but not against Gram-negative bacteria. Generally, the bacteriocins produced from Gram-positive bacteria are active against closely related species and not directly active against Gram-negative bacteria. However, some other bacteriocins from LAB showed antagonistic activities against both Gram-positive and Gram-negative bacteria, like enterocin E760^41^, enterocin E50-52^42^, and salivaricin SMXD51^43^. Owing to the narrow-spectrum capabilities of enterocin OS13α and OS13β against target pathogens, they are favored when compared with conventional broad-spectrum antibiotics that can interfere with the host microbiota, affecting their crucial beneficial roles^22^. Especially during infancy and early childhood, exposure to broad-spectrum antibiotics is often unfavorable, as the early microbiota lacks diversity and stability, rendering it distinctly vulnerable to disturbance, in addition to the importance of intestinal microbiota in early development and education of the immune system^23^.

Noticeably, bacteriocins from isolate OS13 were active against multi-resistant nosocomial enterococci as *Enterococcus faecalis* V583, which was the first VRE *Enterococcus* isolated in the United States, and it was also resistant to high levels of aminoglycosides, macrolides, lincosamides, and streptogramin B^44^. Therefore, BLIS could represent a non-conventional alternative of interest to combat pathogens like enterococci, recognizing that the presence of natural resistance prevents a variety of modern drugs widely used, such as cephalosporins and quinolones, from working effectively against enterococcal infections^45^. Most VREs are also resistant to antibiotics as ampicillin, vancomycin, and aminoglycosides, widely used in the hospital environment. Therefore, only a few therapeutic options are commercially available to treat multidrug-resistant VRE infections, including linezolid, tedizolid, tigecycline, and daptomycin^46,47^. The increased use of these last-source antibiotics and the ability of enterococci to readily acquire new resistance determinants are currently raising the need to find alternative therapeutics and explore the effectiveness of natural antimicrobials such as bacteriocins.

In this study, purification of two BLIS, OS13α and OS13β, from strain OS13 and based on their chromatographic properties exposed that both BLIS were cationic and hydrophobic, which is in agreement with most bacteriocins purified from LAB^48–51^. At each purification level, the antimicrobial activity decreased, especially after reverse-phase chromatography, which was observed in other studies^34,52^. SDS-PAGE did not accurately determine the molecular weight of both BLIS and was unable to differentiate between them^53,54^.

Although both enterocin OS13α and OS13β were heat-labile, and their activity was wholly abolished after heating at 100 ºC for 30 min, their activity together as a crude BLIS in the supernatant was decreased to 6.25% following heat treatment. There are previous reports of many BLIS becoming less heat-stable during subsequent purification steps^55,56^. The larger size of enterocin OS13α and OS13β being 8079.1 Da and 7858.6 Da, respectively, could interpret the reasons behind their heat sensitivity^39,57,58^. Although the crude BLIS with an initial activity of 10,240 AU/mL was decreased to be 1,280 AU/mL (12.5%) after proteinase K treatment but the purified enterocin OS13α was sensitive to proteinase K (50% reduction in its activity), which strongly suggests its proteinaceous nature^52,59^. Also, enterocin OS13β was resistant to proteinase K, and this is probably not due to the absence of digestion sites but more likely due to a tightly folded structure, making it inaccessible to enzymatic digestion^60,61^. The variation in sensitivity of crude BLIS compared to purified ones to proteinase K could indicate that the OS13 isolate might also produce other BLIS besides OS13α and OS13β that is/are more sensitive to proteinase K but was/were not isolated and purified.

Generally, *Enterococcus* spp. found in food are presumed less virulent than clinical isolates^62,63^. Regarding the virulence traits of strain OS13, neither hemolytic activity nor bile salt hydrolase activity was demonstrated, while gelatinase activity was observed. Cytolysin (also called hemolysin) is a lantibiotic, but it differs from most lantibiotics because it is both antibacterial and hemolytic^64^. A higher incidence of cytolysin genes has been shown in clinical isolates (33%) compared with food isolates (6%)^65^. In our study, PCR analysis was used to confirm the absence of the cytolysin gene cluster (*cyl*). Although *E. faecalis* can be an opportunistic pathogen that possesses bile salt hydrolase activity^66^, such activity might be a desirable trait for a probiotic bacterium since it could maximize its ability to survive in the hostile environment of the gastrointestinal tract^67^. The gelatinase enzyme is involved in the virulence of *Enterococcus* sp. and hydrolyzes gelatin, casein, hemoglobin, and other bioactive peptides^65^. The primary role of gelatinase in enterococcal pathogenesis is to provide nutrients to the bacteria by degrading host tissue. However, it also appears to play a role in biofilm formation^68^.

The food chain is considered the primary transmission route of antibiotic-resistant bacteria between populations. *Enterococcus* sp*.* behave as opportunistic pathogens in nosocomial infections and spread antibiotic resistance through the food chain with potential risks to humans^69^. In biosafety evaluations of enterococci, their resistance to glycopeptides, such as vancomycin, should be considered^70^. In our study, the pathogenic potential of the food isolated *E. faecalis* OS13, and the spectrum of resistance to various antibiotics was determined. OS13 was sensitive to ampicillin, penicillin G, vancomycin, erythromycin, kanamycin, and gentamicin. It was resistant to chloramphenicol, streptomycin, tetracycline, clindamycin, fusidic acid, and polymyxin B. These results are in agreement with previous publications^71,72^, where all tested *E. faecalis* strains were sensitive to ampicillin, penicillin, and vancomycin. Resistance to chloramphenicol, tetracycline, erythromycin, gentamicin, and kanamycin is becoming common among enterococci from different sources due to the abuse of antibiotics in clinics, animal and fish farming, and agriculture. Thus, enterococcal antibiotic resistance is not exclusive to the clinical field and is also prevalent in the food industry^71^.

Although MLST is a tool for global epidemiological studies, the possible role of plasmids in transferring virulence between different strains should be considered^73^. MLST analysis of *E. faecalis* OS13 revealed that it belongs to ST116 and CC116. *E. faecalis* with ST116 has been reported as isolated twice from human clinical samples; vancomycin-resistant *E. faecalis* from a blood sample in Denmark and *E. faecalis* DQ287 from a catheter in Cuba^74,75^. To our knowledge, ours is the first report of the isolation of *E. faecalis* with ST116 from non-human samples.

The ability of CC116 to spread in hospitals and cause infection is unknown. However the most common clonal complexes that spread in the hospital environment are CC2, CC6, and CC9^31,76^.

## Conclusion

The food-isolated *E. faecalis* OS13 produced BLIS named enterocin OS13α (8079 Da) and OS13β (7859 Da), with activities against VRE and MDRE. MLST of *E. faecalis* OS13 revealed its belongings to ST116, being the first from non-human samples. *E. faecalis* OS13 was non-hemolytic, bile salt hydrolase-negative, and sensitive to ampicillin, penicillin, vancomycin, erythromycin, kanamycin, and gentamicin, suggesting several safe applications in the food industry and medical treatments. Although our BLIS showed sensitivity to heat treatments, they could be used for preservation of raw or minimally processed foods stored at low temperatures besides their likely role in the treatment of clinical infections with VRE and MDRE. Future works are still required to figure out the amino acid sequences, structures, encoding genes, and mode of actions of such BLIS.

## Supplementary Information

Supplementary Information.
