# Supplementary material for: Purification and characterization of bacteriocins-like inhibitory substances from food isolated Enterococcus faecalis OS13 with activity against nosocomial enterococci
Source: Sci Rep. 2021 Feb 15;11:3795. doi: 10.1038/s41598-021-83357-z (PMC7884432; doi:10.1038/s41598-021-83357-z)
Supplement: Supplementary file 1 — Supplementary Information. [file 41598_2021_83357_MOESM1_ESM.docx]

**Supplementary file**

**Purification and characterization of bacteriocins-like inhibitory substances from food isolated *Enterococcus faecalis* OS13 with activity against nosocomial enterococci**

**Ahmed O. El-Gendy^1*^, Dag A. Brede^2^, Tamer M. Essam^3^, Magdy A. Amin^3^, Shaban H. Ahmed^4^, Helge Holo^2^, Ingolf F. Nes^2^, & Yara I. Shamikh^5,6^**

^1^Microbiology and Immunology Department, Faculty of Pharmacy, Beni-Suef University, Egypt

^2^Department of Chemistry, Biotechnology and Food Science, Laboratory of Microbial Gene Technology and Food Microbiology, Norwegian University of Life Sciences, Norway

^3^Microbiology and Immunology Department and Biotechnology Centre, Faculty of Pharmacy, Cairo University, Egypt

^4^Microbiology and Immunology Department, Faculty of Medicine, Assiut University, Egypt.

^5^Department of Microbiology and Immunology, Nahda University, Beni-Suef, Egypt

^6^Department of Virology, Egypt Center for Research and Regenerative Medicine, Cairo, Egypt

*Corresponding author: Microbiology and Immunology Department, Faculty of Pharmacy, Beni-Suef University, Salah Salem Street, 62511 Beni-Suef, Egypt. Phone: +20 122 347 6015. E-mail: [Ahmed.elgendy@pharm.bsu.edu.eg](mailto:Ahmed.elgendy@pharm.bsu.edu.eg)

**Figure legends**

**Figure S1:** Resistance phenotypes of all bacteriocinogenic isolates to 13 antibiotics. (A) A heatmap representing the resistance phenotypes of each isolate. Both isolates and antibiotics are ordered by hierarchical clustering, reflected by the horizontal and vertical trees, respectively. The heatmap was created in R by the basic heatmap function. Data visualization was done using the R platform version 4.0.3 (https://www.r-project.org) with the heatmap.2 function in the gplots package. (B) Bacteriocinogenic isolates clustering according to their resistance pattern using radial tree generated by the unweighted pair group method with arithmetic mean (UPGMA) method (http://genomes.urv.cat/UPGMA). The tree was drawn by FigTree. (C) A stacked bar plot summarizing the resistance patterns of bacteriocinogenic isolates to 13 antibiotics (Y axis).

**Figure S2:** Distribution of *E. faecalis* (ST 116 and 6 shown in red color) amongst all *E. faecalis* STs included in the MLST database using eBurst v3 program. Each blue circle represents the primary founder or the clonal complex (CC), while each yellow circle represents the subgroup founder or the sequence type (ST) closer to its number. The relative diameter and size of each circle are corresponding to its prevalence amongst isolates in the MLST database. Black lines connect single locus variants (STs that differ in only one of the seven housekeeping genes).

**Figure S3**: The full-length uncropped SDS-PAGE protein marker (i) compared to partially purified bacteriocin after cation exchange chromatography (ii) and showed a single diffused band in the bioassay experiment (iii) with molecular weight in between 7 and 16.5 kDa.

**TABLE S1:** PCR primers used in this study

| Name | Primer | Sequence 5' - 3' | Product size (bp) | Ref. |
| --- | --- | --- | --- | --- |
| 16s rDNA | 11F | TAACACATGCAAGTCGAACG | 1356 | LMGT^*^ |
|  | 4R | ACGGGCGGTGTGTRC |  | LMGT |
| AS-48 | AS-48 F | TTTTTGGGGTTAGCCTTGTT | 191 | ^1^ |
|  | AS-48 R | GCTGCAGCGAGTAAAGAAG |  | ^1^ |
| Bacteriocin 31 | 31 F | TTTGTGGCATTATTGGGATT | 166 | ^2^ |
|  | 31 R | CCATGTTGTACCCAACCATT |  | ^2^ |
| Enterocin A | A F | GACACAACTTATCTATGGGGGTA | 155 | ^3^ |
|  | A R | CTGGAATTGCTCCACCTAAA |  | ^3^ |
| Enterocin B | B F | TGAAACAAATTATCGGTGGAG | 166 | ^4^ |
|  | B R | TATACATTTGCTAACCCAGCAG |  | ^4^ |
| Enterocin L50A & L50B | L50AB F | TTGGGTGGCCTATTGTTAAA | 224 | ^5^ |
|  | L50AB R | TCTATTGTCCATCCTTGTCCA |  | ^5^ |
| Enterocin P | P F | TTTGGTACAAAAGTTGATGCAG | 153 | ^6^ |
|  | P R | ATGTCCCATACCTGCCAAAC |  | ^6^ |
| Enterocin Q | Q F | TTAAAGAAAGGAGGCGGAAA | 107 | ^7^ |
|  | Q R | TGGCAAGCATCCATATTTCA |  | ^7^ |
| Enterolysin A | Lysin A F | CGCAGCTTCTAATGAGTGGT | 161 | ^8^ |
|  | Lysin A R | CATACACACTGCCATTTCCA |  | ^8^ |
| Mundticin | Mund F | AACAGCAAAAGAAATGTCACAA | 154 | ^9^ |
|  | Mund R | ACCAGCTGCTCCACCAGTA |  | ^9^ |
| Cytolysin | Cyto F | TGGCGGTATTTTTACTGGAG | 186 | ^10^ |
|  | Cyto R | TGAATCGCTCCATTTCTTC |  | ^10^ |
| Bacteriocin 32 | 32 F | AGCTTTCACCCCTTCTGTTTC | 105 | This study |
|  | 32 R | GGCACTTTAGCACCTTTTGG |  | This study |
| Bacteriocin GM-1 | GM-1 F | ACGCGTTCATATGGTAATGG | 129 | This study |
|  | GM-1 R | ATGTCCCATACCTGCCAAAC |  | This study |
| Enterocin SE-K4 | SE-K4 F | GCATGATTGGGATTGGTTTT | 150 | This study |
|  | SE-K4 R | CGTATGCTTTTACGCCTCTG |  | This study |
| Bacteriocin T8 | T8 F | TTGTCTAGCTGGCATCGGTA | 156 | This study |
|  | T8 R | GGACCATGATTAACCCAACC |  | This study |
| MR10A MR10B | MR10AB F | AGTTTGGATGGCCAATTGTT | 223 | This study |
|  | MR10AB R | TGTCCATCCTTGTCCGATAAA |  | This study |
| Enterocin 1071 A & B | 1071 AB F | AGGTCCAGCTGCTTATTGGA | 202 | This study |
|  | 1071 AB R | TTCCAGGTCCTCCACCAGTA |  | This study |
| Enterocin EJ97 | EJ97 F | TGTTAGCAAAAATTAAAGCGATGA | 118 | This study |
|  | EJ97 R | TCCCAAGGATAACGACCGTA |  | This study |
| Glucose-6-phosphate dehydrogenase | gdh-1 | GGCGCACTAAAAGATATGGT | 530 | ^11^ |
|  | gdh-2 | CCAAGATTGGGCAACTTCGTCCCA |  | ^11^ |
| Glyceraldehydes-3-phosphate dehydrogenase | gyd-1 | CAAACTGCTTAGCTCCAATGGC | 395 | ^11^ |
|  | gyd-2 | CATTTCGTTGTCATACCAAGC |  | ^11^ |
| Phosphate ATP binding cassette transporter | pstS-1 | CGGAACAGGACTTTCGC | 583 | ^11^ |
|  | pstS-2 | ATTTACATCACGTTCTACTTGC |  | ^11^ |
| Glucokinase | gki-1 | GATTTTGTGGGAATTGGTATGG | 438 | ^11^ |
|  | gki-2 | ACCATTAAAGCAAAATGATCGC |  | ^11^ |
| Shikimate-5-dehydrogenase | aroE-1 | TGGAAAACTTTACGGAGACAGC | 459 | ^11^ |
|  | aroE-2 | GTCCTGTCCATTGTTCAAAAGC |  | ^11^ |
| Xanthine phosphoribosyltransferase | xpt-1 | AAAATGATGGCCGTGTATTAGG | 456 | ^11^ |
|  | xpt-2 | AACGTCACCGTTCCTTCACTTA |  | ^11^ |
| Acetyl-CoA acetyltransferase | yiqL-1 | CAGCTTAAGTCAAGTAAGTGCCG | 436 | ^11^ |
|  | yiqL-2 | GAATATCCCTTCTGCTTGTGCT |  | ^11^ |

* LMGT: Laboratory of Microbial Gene Technology, Norwegian University of Life Sciences, Norway.

**TABLE S2:** MICs and antibiotics sensitivity test of bacteriocins producing *E.faecalis* isolates

| **Antibiotic ^a^** | **Range of tested Conc.**  **µg/mL** | **Break-points ^b^ of *E. faecalis***  **µg/ml** | **Sensitivity ^c^ / MIC ^d^** | | | | | | | | | | | |
| --- | --- | --- | --- | --- | --- | --- | --- | --- | --- | --- | --- | --- | --- | --- |
|  |  |  |  | **OS7** | **OS11** | **OS13** | **OS16** | **OS28** | **OS29a** | **OS29b** | **OS62a** | **OS62b** | **OS64** | **OS65** |
| **AMP.** | 32 – 0.25 | 16 |  | (S) / 4 | (S) / 2 | (S) / 2 | (S) / 2 | (S) / 4 | (S) / 4 | (S) / 4 | (S) / 4 | (S) / 4 | (S) / 2 | (S) / 4 |
| **APR.** | 1024 – 8 | 512 |  | (R) / 512 | (R) / 512 | (R) / 512 | (R) / 512 | (R) / 512 | (R) / 512 | (R) / 512 | (R) / 512 | (R) / 512 | (R) / 512 | (R) / 512 |
| **CM.** | 128 – 1 | 32 |  | (S) / 4 | (R) / 64 | (R) / 64 | (R) / 64 | (S) / 4 | (S) / 4 | (S) / 4 | (S) / 4 | (R) / 64 | (R) / 64 | (S) / 4 |
| **CLIND.** | 32 – 0.25 | 4 |  | (R) / >32 | (R) / >32 | (R) / >32 | (R) / >32 | (R) / >32 | (R) / >32 | (R) / >32 | (R) / >32 | (R) / >32 | (R) / >32 | (R) / >32 |
| **E.** | 64 – 0.5 | 8 |  | (R) / ≥64 | (S) / 4 | (S) / 4 | (S) / 4 | (R) / ≥64 | (R) / ≥64 | (R) / ≥64 | (R) / ≥64 | (R) / ≥64 | (S) / 2 | (R) / ≥64 |
| **FUS.** | 64 – 0.5 | 2 |  | (R) / 4 | (R) / 4 | (R) / 4 | (R) / 4 | (R) / 4 | (R) / 4 | (R) / 4 | (R) / 4 | (R) / 4 | (R) / 4 | (R) / 4 |
| **GEN.** | 2048 – 16 | 1024 |  | (R) />2048 | (S) / 32 | (S) / 32 | (S) / 32 | (R) />2048 | (R) />2048 | (R) />2048 | (R) />2048 | (R) />2048 | (S) / 32 | (R) />2048 |
| **KAN.** | 4096 – 32 | 512 |  | (R) />4096 | (S) / 64 | (S) / 64 | (S) / 64 | (R) />4096 | (R) />4096 | (R) />4096 | (R) />4096 | (R) />4096 | (S) / 64 | (R) />4096 |
| **PEN.** | 32 – 0.25 | 16 |  | (R) / 16 | (S) / 4 | (S) / 4 | (S) / 4 | (R) / 16 | (R) / 16 | (R) / 16 | (R) / 16 | (R) / 16 | (S) / 4 | (R) / 16 |
| **PB.** | 256 – 2 | 4 |  | (R) />256 | (R) />256 | (R) />256 | (R) />256 | (R) />256 | (R) />256 | (R) />256 | (R) />256 | (R) />256 | (R) />256 | (R) />256 |
| **STR.** | 8192 – 64 | 2048 |  | (R) />8192 | (R) / 2048 | (R) / 2048 | (R) / 2048 | (R) />8192 | (R) />8192 | (R) />8192 | (R) />8192 | (R) />8192 | (R) / 2048 | (R) />8192 |
| **TET.** | 128 – 1 | 16 |  | (R) / 32 | (R) / 64 | (R) / 64 | (R) / 64 | (R) / 32 | (R) / 32 | (R) / 32 | (R) / 32 | (R) / 32 | (R) / 128 | (R) / 32 |
| **VAN.** | 64 – 0.5 | 32 |  | (S) / 1 | (S) / 1 | (S) / 1 | (S) / 1 | (S) / ≤0.5 | (S) / ≤0.5 | (S) / 1 | (S) / ≤0.5 | (S) / 1 | (S) / 1 | (S) / ≤0.5 |

^a^ Antibiotics abbreviations: Ampicillin, AMP; Apramycin, APR; Chloramphenicol, CM; Clindamycin, CLIND; Erythromycin, E; Fusidic acid, FUS; Gentamicin, GEN; Kanamycin, KAN; Penicillin G, PEN; Polymyxin B, PB; Streptomycin, STR; Tetracyclin, TET; Vancomycin, VAN.

^b^ A breakpoint is a concentration of an antibiotic which indicates whether the bacteria is susceptible or resistant to the antibiotic. If the MIC is less than or equal to the susceptibility breakpoint the bacteria is considered susceptible to the antibiotic.

^c^ S, Sensitive; R, Resistant

^d^ MIC (µg/mL), Minimum Inhibitory concentration.

**TABLE S3:** Multilocus sequence typing (MLST) of putative bacteriocinogenic *E. faecalis* isolates

| **NCBI accession numbers of partial 16s rRNA gene (Present study)** | **Strain** | **Source** | **Allelic Profile ^a^** | | | | | | | **ST ^b^** | **CC ^c^** |
| --- | --- | --- | --- | --- | --- | --- | --- | --- | --- | --- | --- |
|  |  |  | ***gdh*** | ***gyd*** | ***pstS*** | ***gki*** | ***aroE*** | ***xpt*** | ***yiqL*** |  |  |
| JX536093 | OS7 | Stool | 12 | 7 | 3 | 7 | 6 | 1 | 5 | 6 | 6 |
| JX536097 | OS11 | Food | 17 | 2 | 22 | 1 | 14 | 14 | 1 | 116 | 116 |
| JQ889271 | OS13 | Food | 17 | 2 | 22 | 1 | 14 | 14 | 1 | 116 | 116 |
| JX536104 | OS16 | Food | 17 | 2 | 22 | 1 | 14 | 14 | 1 | 116 | 116 |
| JX536118 | OS28 | Urine ♂ | 12 | 7 | 3 | 7 | 6 | 1 | 5 | 6 | 6 |
| JX536119 | OS29a | Urine ♂ | 12 | 7 | 3 | 7 | 6 | 1 | 5 | 6 | 6 |
| N/S^d^ | OS29b | Urine ♂ | 12 | 7 | 3 | 7 | 6 | 1 | 5 | 6 | 6 |
| N/S | OS62a | Urine ♂ | 12 | 7 | 3 | 7 | 6 | 1 | 5 | 6 | 6 |
| N/S | OS62b | Urine ♂ | 12 | 7 | 3 | 7 | 6 | 1 | 5 | 6 | 6 |
| N/S | OS64 | Stool | 17 | 2 | 22 | 1 | 14 | 14 | 1 | 116 | 116 |
| JX536120 | OS65 | Urine ♀ | 12 | 7 | 3 | 7 | 6 | 1 | 5 | 6 | 6 |

^a^ The allelic profile of 7 housekeeping genes: *gdh*, glucose-6-phosphate dehydrogenase; *gyd*, glyceraldehydes-3-phosphate dehydrogenase; *pstS*, phosphate ATP binding cassette transporter; *gki*, glucokinase; *aroE*, shikimate-5-dehydrogenase; *xpt*, xanthine phosphoribosyltransferase; *yiqL*, acetyl- CoA acetyltransferase.

^b^ ST, Sequence Type.

^c^ CC, Clonal Complex.

^d^ N/S, not submitted

**Figure S1**

**(B)**

**(A)**

| 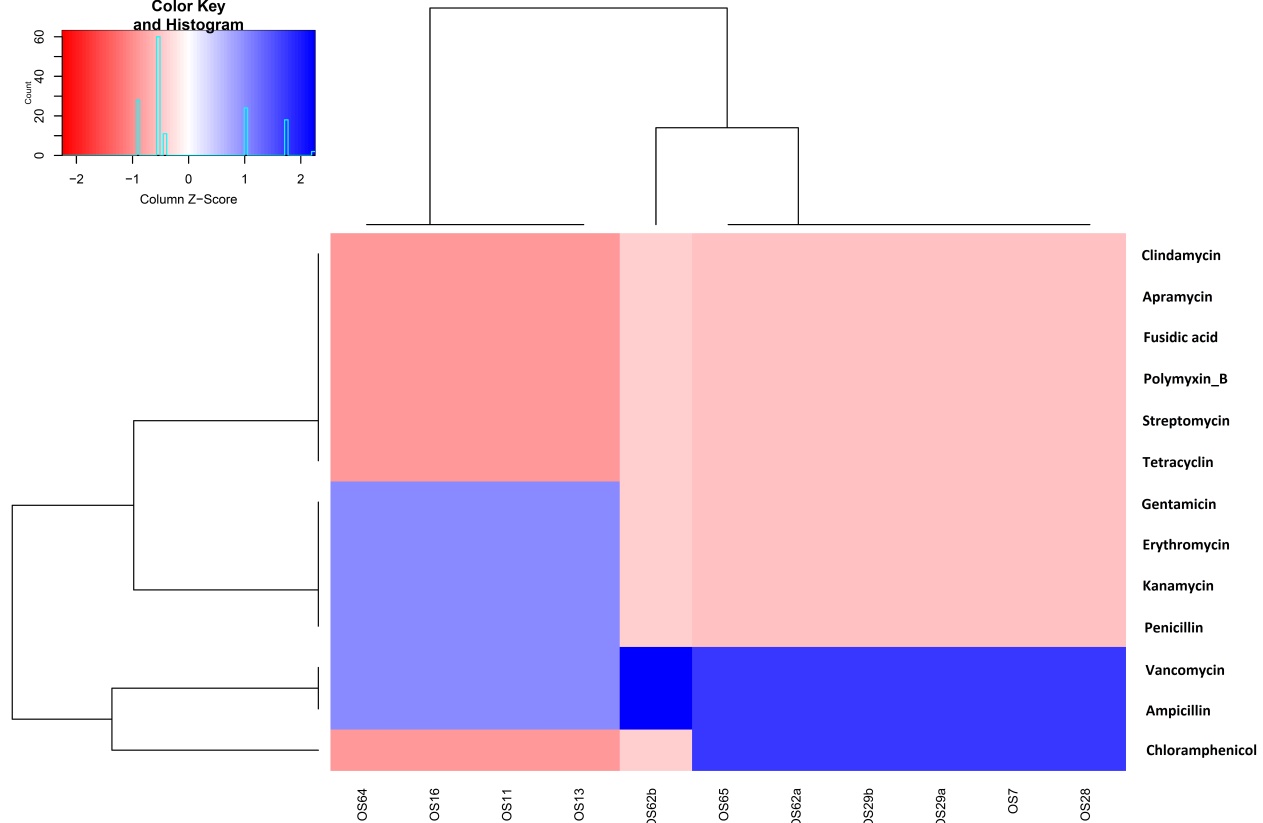 | 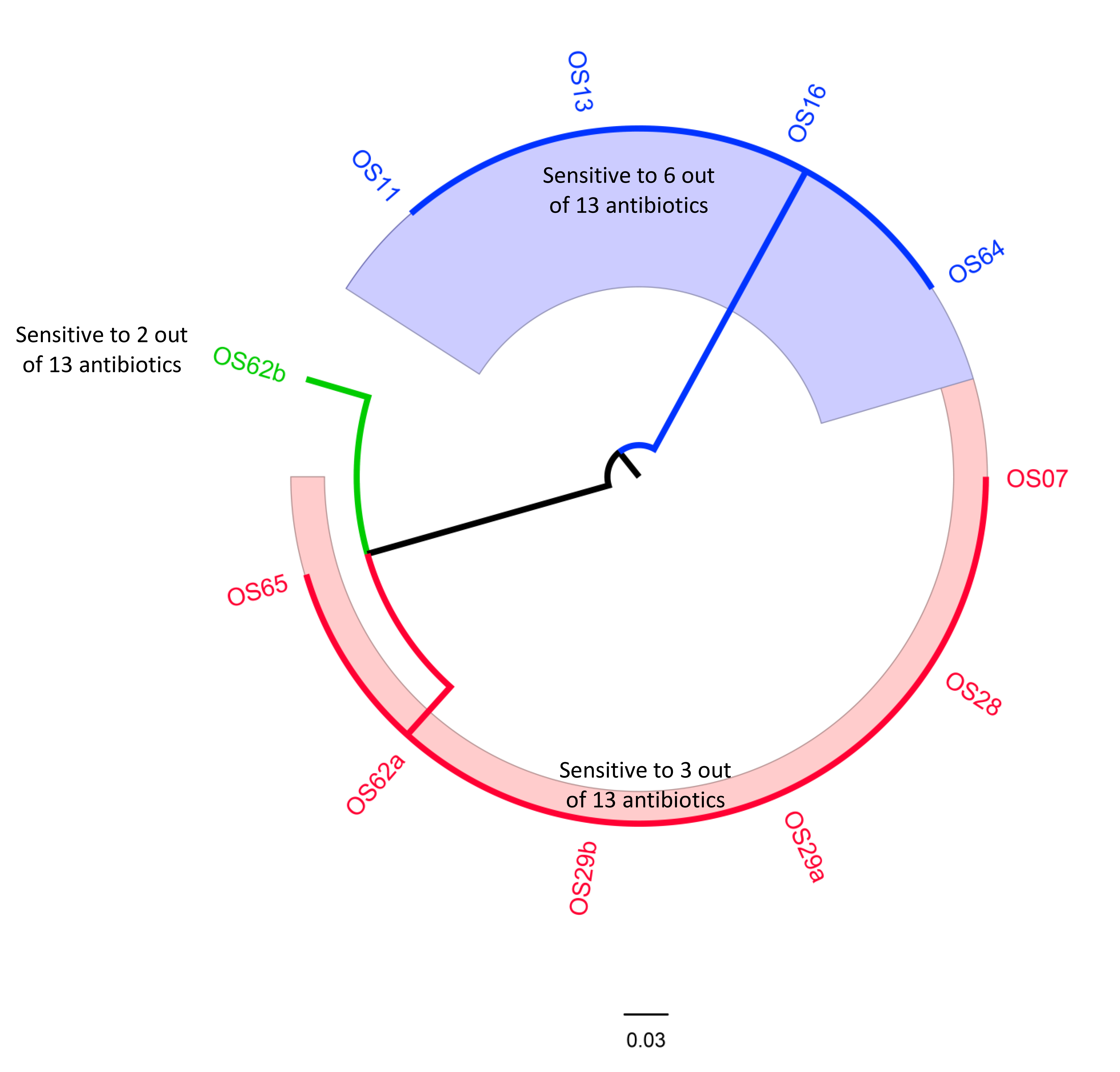 |
| --- | --- |
| **(C)**  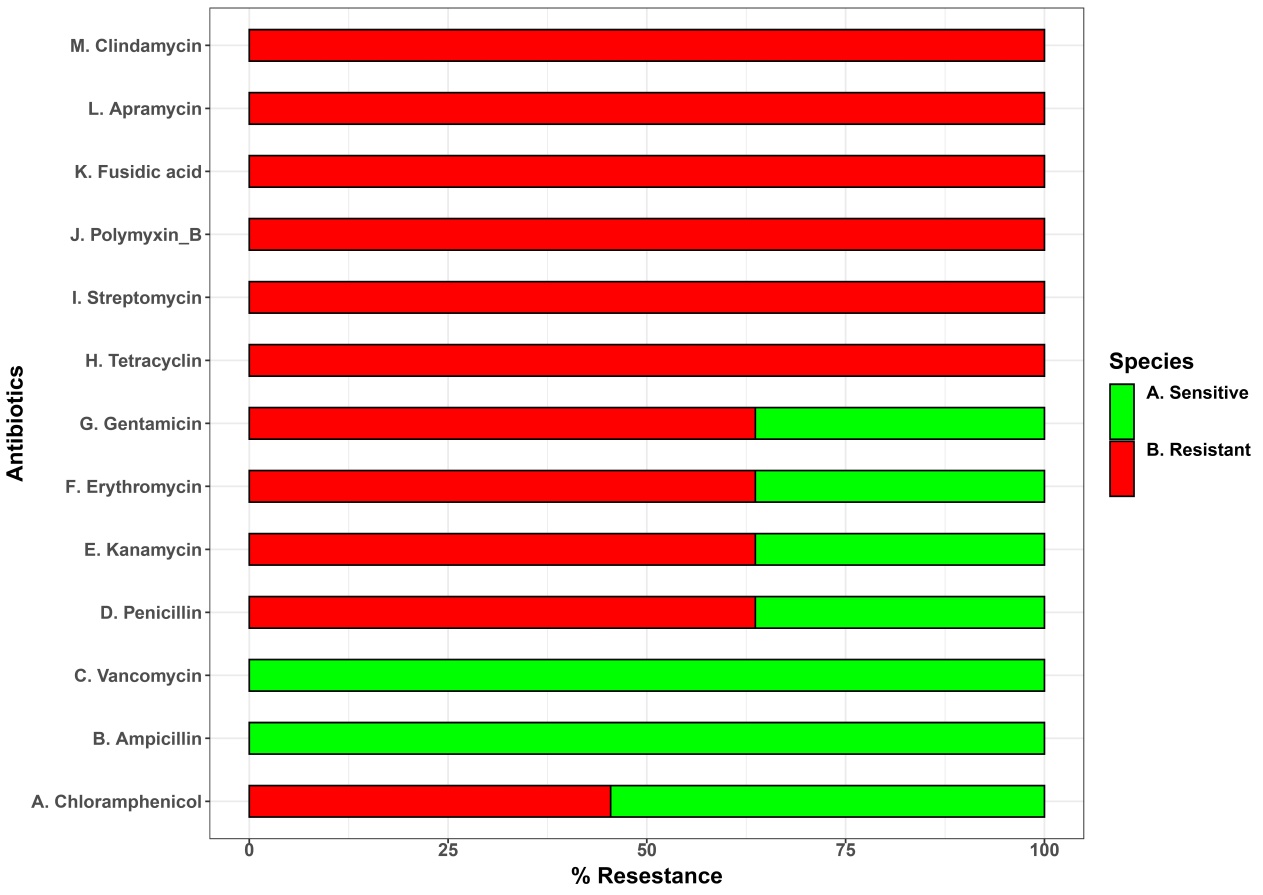 | |

**Figure S2**


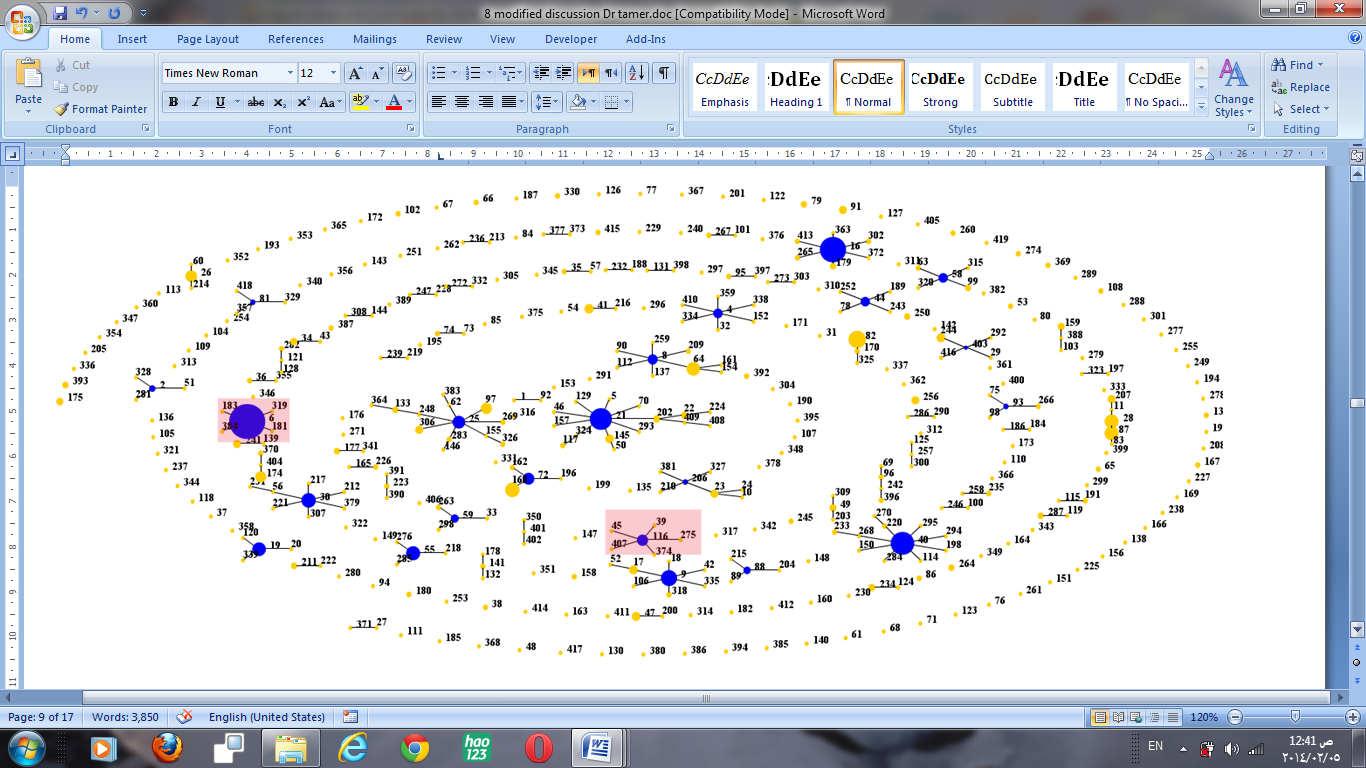


**\**

**Figure S3**

| **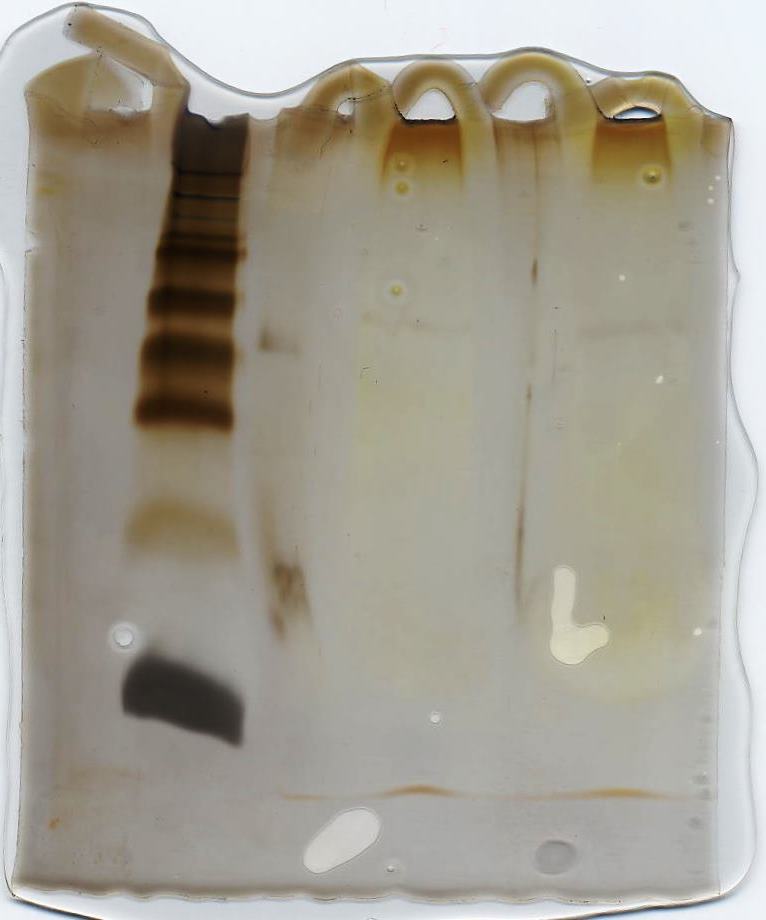**  25 KDa  16.5 KDa  7 KDa | | **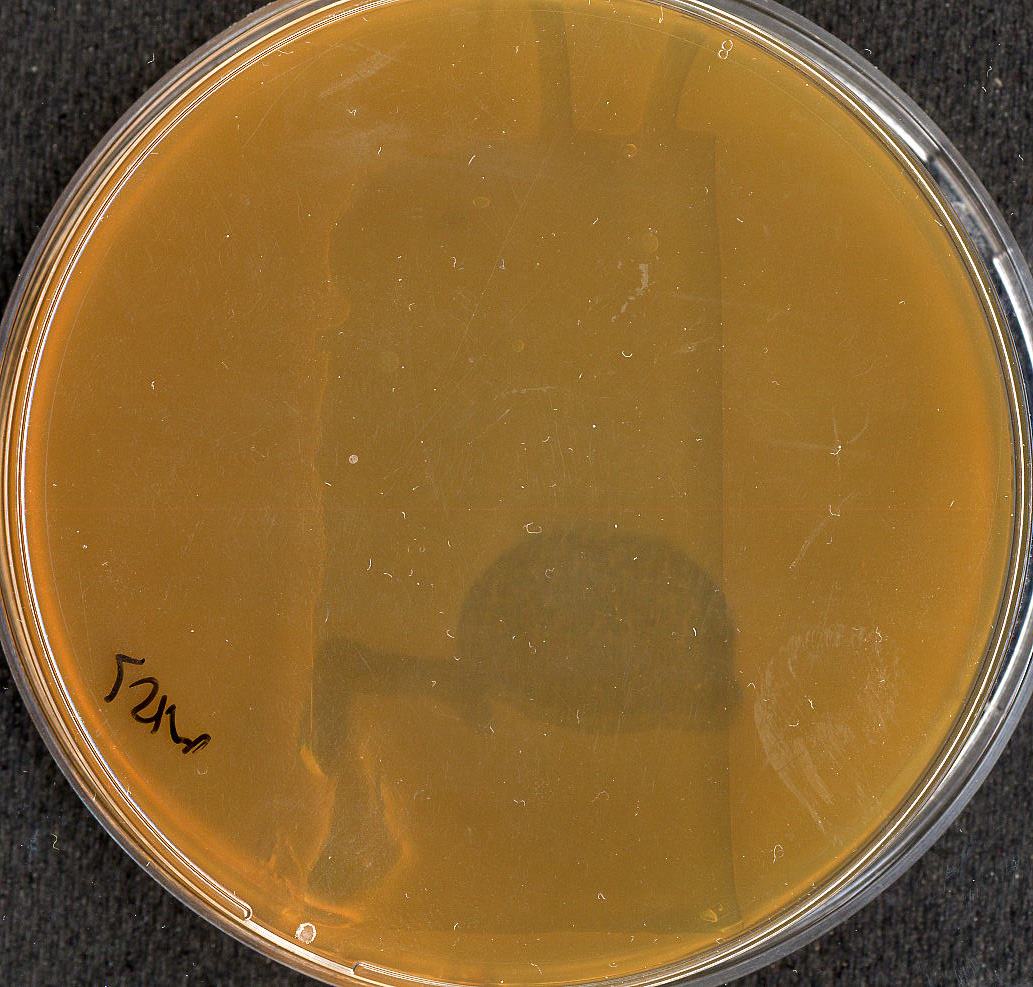** |
| --- | --- | --- |
| **(i)** | **(ii)** | **(iii)** |

**REFERENCES**:

1 Martínez-Bueno, M. *et al.* Determination of the gene sequence and the molecular structure of the enterococcal peptide antibiotic AS-48. *J Bacteriol* **176**, 6334-6339, doi:10.1128/jb.176.20.6334-6339.1994 (1994).

2 Tomita, H., Fujimoto, S., Tanimoto, K. & Ike, Y. Cloning and genetic organization of the bacteriocin 31 determinant encoded on the Enterococcus faecalis pheromone-responsive conjugative plasmid pYI17. *J Bacteriol* **178**, 3585-3593, doi:10.1128/jb.178.12.3585-3593.1996 (1996).

3 Aymerich, T. *et al.* Biochemical and genetic characterization of enterocin A from Enterococcus faecium, a new antilisterial bacteriocin in the pediocin family of bacteriocins. *Appl Environ Microbiol* **62**, 1676-1682, doi:10.1128/aem.62.5.1676-1682.1996 (1996).

4 Franz, C. M. *et al.* Atypical genetic locus associated with constitutive production of enterocin B by Enterococcus faecium BFE 900. *Appl Environ Microbiol* **65**, 2170-2178, doi:10.1128/aem.65.5.2170-2178.1999 (1999).

5 Cintas, L. M. *et al.* Enterocins L50A and L50B, two novel bacteriocins from Enterococcus faecium L50, are related to staphylococcal hemolysins. *J Bacteriol* **180**, 1988-1994, doi:10.1128/jb.180.8.1988-1994.1998 (1998).

6 Cintas, L. M., Casaus, P., Håvarstein, L. S., Hernández, P. E. & Nes, I. F. Biochemical and genetic characterization of enterocin P, a novel sec-dependent bacteriocin from Enterococcus faecium P13 with a broad antimicrobial spectrum. *Appl Environ Microbiol* **63**, 4321-4330, doi:10.1128/aem.63.11.4321-4330.1997 (1997).

7 Cintas, L. M. *et al.* Biochemical and genetic evidence that Enterococcus faecium L50 produces enterocins L50A and L50B, the sec-dependent enterocin P, and a novel bacteriocin secreted without an N-terminal extension termed enterocin Q. *J Bacteriol* **182**, 6806-6814, doi:10.1128/jb.182.23.6806-6814.2000 (2000).

8 Nilsen, T., Nes, I. F. & Holo, H. Enterolysin A, a cell wall-degrading bacteriocin from Enterococcus faecalis LMG 2333. *Appl Environ Microbiol* **69**, 2975-2984, doi:10.1128/aem.69.5.2975-2984.2003 (2003).

9 Bennik, M. H., Vanloo, B., Brasseur, R., Gorris, L. G. & Smid, E. J. A novel bacteriocin with a YGNGV motif from vegetable-associated Enterococcus mundtii: full characterization and interaction with target organisms. *Biochimica et biophysica acta* **1373**, 47-58, doi:10.1016/s0005-2736(98)00086-8 (1998).

10 Gilmore, M. S. *et al.* Genetic structure of the Enterococcus faecalis plasmid pAD1-encoded cytolytic toxin system and its relationship to lantibiotic determinants. *J Bacteriol* **176**, 7335-7344, doi:10.1128/jb.176.23.7335-7344.1994 (1994).

11 Ruiz-Garbajosa, P. *et al.* Multilocus sequence typing scheme for Enterococcus faecalis reveals hospital-adapted genetic complexes in a background of high rates of recombination. *Journal of clinical microbiology* **44**, 2220-2228, doi:10.1128/jcm.02596-05 (2006).
